# Neuropathic pain: A patient‐centred approach to measuring outcomes

**DOI:** 10.1111/hex.12673

**Published:** 2018-04-15

**Authors:** Steve Hwang, Floortje van Nooten, Ted Wells, Aisling Ryan, Bruce Crawford, Christopher Evans, Marci English

**Affiliations:** ^1^ Endpoint Outcomes Boston MA USA; ^2^ Astellas Pharma B.V. Leiden Netherlands; ^3^ IMS Japan K.K. Tokyo Japan; ^4^ Astellas Pharma Inc. Northbrook IL USA; ^5^ Present address: Dompé Farmaceutici S.p.A. Milan Italy; ^6^ Present address: Bruce Crawford, inVentiv Health Tokyo Japan

**Keywords:** diabetic neuropathies, neuralgia, pain, post‐herpetic neuralgia, qualitative research, quality of life

## Abstract

**Background:**

Neuropathic pain (NP) is a complex, chronic pain state initiated by a primary lesion or dysfunction of the nervous system and presents as a variety of symptoms across multiple disease states.

**Objective:**

To develop a patient‐centred conceptual model of symptoms and impacts in subjects with diabetic peripheral neuropathy (DPN) or post‐herpetic neuralgia (PHN) that can inform the measurement strategy in clinical trials.

**Method:**

Thirty subjects with DPN or PHN participated in in‐person interviews which were performed until saturation was achieved. Transcripts were analysed in ATLAS.ti.

**Results:**

Interviews were completed with DPN subjects (United States, n = 10; Japan, n = 10) and PHN subjects (United States, n = 5; Japan, n = 5). Numbness and tingling were frequently reported symptoms in the DPN population while itchiness and hypersensitivity were predominant in PHN. Both populations experienced burning and ache/soreness with similar frequency. DPN subjects experienced pain primarily in their lower extremity (eg feet, ankles), while PHN subjects experienced pain primarily in the chest and back. Impacts reported by DPN subjects included difficulty walking, sleep disturbance and climbing stairs. Impacts in PHN subjects included sleep disturbance, avoidance of physical contact, being angry/frustrated and being sad/depressed. Overall, concepts in Japan were not qualitatively different from the United States. Conceptual models of NP were generated based on the concepts elicited.

**Conclusions:**

This research highlights core concepts to measure from the patient's perspective. Moreover, it enables the assessment of existing measures, the possible modification of these measures, or if a new NP measure with improved sensitivity and responsiveness is merited.

## 1 INTRODUCTION

The International Association for the Study of Pain defines neuropathic pain as “pain arising as a direct consequence of a lesion or disease affecting the somatosensory system.”^1^ It is typically accompanied by tissue injury and presents itself with a variety of symptoms across multiple disease states.^2^ Diabetic peripheral neuropathy (DPN) and post‐herpetic neuralgia (PHN) are 2 common diseases characterized as having a neuropathic pain component. In the literature, the quality of neuropathic pain in DPN and PHN is commonly described as burning pain, numbness, electric shock‐like sensation and tingling or skin‐crawling sensation.^3^ The pain can be spontaneous, constant or triggered in response to a stimuli (eg movement or touch).^4^

Despite the high prevalence of DPN and PHN, the mechanism of neuropathic pain is complex and treatment remains a challenge. Neuropathic pain is largely underreported and undertreated with best prevalence estimates between 7% and 10%^5^ in the general population, while conservative estimates place prevalence at 1%‐2%.^6^ Twenty‐six to 47% of people who suffer from diabetes mellitus experience DPN, whereas 25%‐50% of people who suffer from herpes zoster viral disorder will develop PHN, depending on timing of antiviral therapy for herpes zoster.^7^ Despite its prevalence, many management strategies for neuropathic pain are suboptimal and it remains an area of largely unmet therapeutic need.^8^ Treatments include antidepressants (eg duloxetine), anticonvulsants (eg gabapentin, pregabalin), local anaesthetics (eg lidocaine patch, mexiletine, topical capsaicin) and opioid analgesics (eg tramadol)^9^ with polypharmacy often used.^10^ Patients have reported feeling dissatisfied with the efficacy of management therapies,^10^, ^11^ and the financial burden caused by direct medical costs, hospitalizations and outpatient visits, loss of the ability to work, loss of a caregivers’ ability to work and possibly greater need for institutionalization or other living assistance.^12^, ^13^, ^14^ Furthermore, patients may resort to self‐management using non‐approved alternative therapies or may learn to accept and live with the pain, adjusting their lifestyle as needed.^15^

Neuropathic pain significantly impacts the daily lives of patients with DPN or PHN and is often associated with psychological comorbidities (ie depression and anxiety), sleep disturbance and detriments to self‐confidence and self‐esteem.^13^, ^14^, ^16^, ^17^ Impacts experienced by patients with DPN are reported as decreased physical functioning, mobility, home productivity, lack of energy and vitality, poor sleep quality, limitations in social relationships and enjoyment of life.^16^ Similarly, patients with PHN associate their pain with fatigue, insomnia, depression, anxiety, interference with social roles and leisure activity.^7^ Across aetiologies, neuropathic pain significantly interferes with physical activity, role and social functions, and negatively affects overall health‐related quality of life (HRQoL).^2^, ^14^, ^16^, ^18^

There are substantial societal costs associated with neuropathic pain including direct and indirect costs and caregiver costs.^6^, ^12^ Patients with DPN incur substantial health‐care costs due to outpatient visits and hospitalizations, and humanistic burden increases as pain severity increases,^16^, ^18^, ^19^ a scenario also observed in PHN.^11^ In Western Europe, direct medical costs have been shown to be twice as high as a control group of chronic pain patients.^13^ A patient‐centric approach to drug development, where patient needs and preferences are aligned with decision making, is becoming increasingly important to provide cost‐effective treatment options.^20^

In designing the measurement strategy of clinical studies, patient involvement in the form of qualitative interviews is encouraged by the Food and Drug Administration's (FDA) as evident in their 2009 Patient‐Reported Outcome (PRO) Guidance. Previous primary research with patients includes a multicountry focus group study in which the content validity of the Neuropathic Pain Symptom Inventory (NPSI) was explored;^21^ however, that research was conducted before the FDA's 2009 PRO Guidance and is limited to assessing the validity of a single, symptom‐focused measure. This research expands on that investigation, with the goal of examining the impacts of neuropathic pain in addition to symptoms and seeks to develop an overarching conceptual model that can be compared to existing measures and explore where gaps lie in current measures.

## 2 MATERIALS AND METHODS

### 2.1 Ethics

Study documents were submitted to Copernicus Group Independent Review Board for ethics review and approval. In Japan, local ethics board approval was obtained. Research practices were guided by the Good Clinical Practice and regulatory requirements as applicable.

### 2.2 Recruitment

Subjects were recruited from the United States and Japan, with a target population of 15 subjects with DPN and 15 subjects with PHN (5 subjects within each group were targeted in Japan). Clinicians were asked to attempt to recruit subjects with differing ages, genders and ethnicities (ethnicity only applicable to the United States).

Clinicians used existing medical records and the pre‐specified inclusion/exclusion criteria to identify potentially eligible subjects. Informed consent was obtained from the subject prior to participation, and the case report form was completed to document the subject's eligibility. Subjects also completed a basic demographic information form. All documents were reviewed to confirm subject eligibility and correct completion prior to conducting the interviews.

### 2.3 Inclusion/exclusion criteria

Subjects recruited for the study were only enrolled if they satisfied the following inclusion criteria: 18 years of age or older; a confirmed diagnosis with DPN or PHN; an average daily pain score ≥4 on an 11‐point 0‐10 numerical rating scale at time of screening; in good general and psychological health and capable of completing a 60‐minute face‐to‐face interview; able to speak, read, write and comprehend US English (US subjects) or Japanese (Japanese subjects). In addition, subjects with DPN were required to have an established diagnosis of diabetes (Type 1 or Type 2) with painful DPN and at least a 1‐year history of DPN pain. Subjects with PHN were required to have had pain present ≥6 months after healing of their herpes zoster rash.

Exclusion criteria included as follows: presence of significant pain of an aetiology other than DPN or PHN (eg compression‐related neuropathies [eg spinal stenosis, fibromyalgia or arthritis]) that may have interfered with the assessment of DPN‐ or PHN‐related pain; presence of a cognitive impairment or psychiatric condition that would have interfered with the subject's ability to complete a questionnaire and effectively participate in a 60‐minute interview; the presence of any condition which, in the investigator's opinion, made the subject unsuitable for study.

### 2.4 Concept elicitation interviews

Trained interviewers obtained verbal consent from the subject prior to audio‐recording and conducting the 60‐minute face‐to‐face interview (see Supporting Information for interview questions). Subjects were asked open‐ended questions designed to encourage spontaneous reporting of symptoms and impacts. Interviewers only probed as necessary if any concepts required clarification or additional context. Probed concepts were not part of evaluating saturation because saturation is assessed by documenting concept emergence. After the first 25% of interviews were completed, interviewers discussed the findings at that stage and made adjustments to the interview process as needed. Subjects who participated in interviews were compensated for their time and travel costs.

### 2.5 Transcription/translation and analysis

The audio recordings of the interviews were transcribed verbatim and analysed for content using the qualitative data analysis software, ATLAS.ti. Transcripts of interviews with US subjects were analysed by native English speakers, and transcripts with Japanese subjects were analysed by native Japanese speakers. Concepts identified during analysis of Japanese interviews were translated to English for comparison with US subjects.

The coding process was guided by established qualitative research methods, including grounded theory and constant comparative method. In grounded theory, inductive, yet systematic analytic strategies are applied to qualitative data to conceptually analyse individual experiences.^22^, ^23^ Coding schemes were developed to identify thematic trends in subject descriptions of symptoms and impacts related to their neuropathic pain experience. There were 3 coders, and harmonization meetings were held periodically (ie after first transcript, after 10 transcripts) to compare, reconcile and update the thematic coding scheme. The wording of the codes was harmonized to be conceptually equivalent and accurate (eg “feeling hot to the touch,” “hot pain” and “burning pain” consolidated to “burning”). The concepts and domains identified, along with examples of subject quotes, were compared across aetiologies and subject populations and informed the development of a conceptual model in DPN and PHN.

### 2.6 Saturation

Saturation is considered to be achieved at the point when additional interviews are unlikely to yield new information (ie new concepts of importance and relevance to subjects).^22^ To evaluate conceptual saturation, concepts spontaneously emerging from the interviews were documented per subject, constantly comparing the total number of concepts that have already emerged from the previous subject(s) to the subsequent subject. Concepts were also compared in sets, in the order that data were collected. An example would be a group of 10 subjects, where the first set of interviews (eg n = 3) are compared with the next set (eg n = 4). Both of these sets of interviews (eg n = 7) are then compared with the final set of interviews (eg n = 3). The goal of this process is to compare the amount of novel information that is observed in the first interview set compared with the second interview set and so forth. A low number of subjects reporting a certain concept are neither an indication of whether saturation is achieved or not achieved. Instead, the timing of the concept occurrence during the interview process determines whether there is adequate evidence of saturation.

Saturation in the US population was evaluated and then compared to the Japanese population to determine if saturation was achieved in the total sample. In addition to confirming the adequacy of the sample size, this process highlights the emergence of new concepts to develop a comprehensive list of concepts, as well as the emergence of subconcepts that will help to saturate broader concepts.

## 3 RESULTS

### 3.1 Demographics

Table 1 summarizes demographic information for all subjects with DPN and PHN who participated in interviews, respectively.

**Table 1:** Patient demographic and health information

Characteristic	DPN		PHN	
	US (N = 10) N (%)	Japan (N = 5) N (%)	US (N = 9) N (%)	Japan (N = 5) N (%)
Age				
41‐50	2 (20.0)	1 (20.0)	1 (11.1)	0 (0.0)
51‐60	0 (0.0)	2 (40.0)	3 (33.3)	0 (0.0)
61‐70	4 (40.0)	1 (20.0)	2 (22.2)	1 (20.0)
71+	4 (40.0)	1 (20.0)	3 (33.3)	4 (80.0)
Gender				
Female	4 (40.0)	1 (20.0)	4 (44.4)	3 (60.0)
Male	6 (60.0)	4 (80.0)	5 (55.6)	2 (40.0)
Race				
White or Caucasian	10 (100.0)	0 (0.0)	5 (55.6)	0 (0.0)
Asian	0 (0.0)	5 (100.0)	2 (22.2)	0 (0.0)
Other	0 (0.0)	0 (0.0)	2 (22.2)^a^	0 (0.0)
Ethnicity				
Hispanic/Latino	1 (10.0)	0 (0.0)	2 (22.2)	0 (0.0)
Education				
High school diploma (or GED) or less	4 (40.0)	0 (0.0)	5 (55.6)	5 (100.0)
Some college or certificate programme	5 (50.0)	0 (0.0)	3 (33.3)	0 (0.0)
College or university degree (2‐ or 4‐year)	1 (10.0)	4 (80.0)	1 (11.1)	0 (0.0)
Did not specify	0 (0.0)	1 (20.0)	–	–
Living status				
With husband/wife/partner	7 (70.0)	1 (20.0)	3 (33.3)	4 (80.0)
Alone	2 (20.0)	0 (0.0)	5 (55.6)	0 (0.0)
With partner and children	5 (25.0)	3 (60.0)		
With parents	1 (10.0)	0 (0.0)		
Did not specify	0 (0.0)	1 (20.0)		
Other			1 (11.1)^b^	1 (5.0)^c^
Work status^d^				
On disability	1 (10.0)	3 (30.0)	5 (55.6)	0 (0.0)
Part time	1 (10.0)	1 (20.0)	4 (44.4)	3 (30.0)
Full time	2 (20.0)	2 (40.0)	2 (22.2)	0 (0.0)
Retired	5 (50.0)	0 (0.0)	0 (0.0)	1 (5.0)
Unemployed	1 (10.0)	1 (20.0)	0 (0.0)	4 (80.0)
Aetiology				
Type 1 diabetes	1 (10.0)	0 (0.0)	–	–
Type 2 diabetes	9 (90.0)	5 (100.0)	–	–

DPN, diabetic peripheral neuropathy; GED, General Education Development; PHN, post‐herpetic neuralgia; US, United States.

^a^Hispanic; Mexican‐American; ^b^With child; ^c^With partner, children and parents; ^d^Some patients marked more than one response, thus percentages in each category may total more than 100.0% for both Japanese and US populations.

### 3.2 US study population

Nine US subjects had a diagnosis of Type 2 diabetes (90.0%) and 1 subject had Type 1 diabetes (10.0%). The majority of US subjects with DPN were >60 years old (80.0%), male (n = 6, 60.0%), and all 10 were Caucasian (100.0%), with 1 subject identifying as Hispanic or Latino as well (n = 1, 10.0%).

Ten US subjects with PHN participated in the concept elicitation interviews. However, during the interview, 1 subject revealed that neuropathic pain only occurred during herpetic episodes, which resulted in removal of this subject during data analysis. US subjects with PHN were above the age of 41, with 3 subjects between 51 and 60 years old (n = 3, 33.3%) and 3 above the age of 71 (n = 3, 33.3%). The gender ratio was near equal, with 5 male subjects (n = 5, 55.6%) and approximately half of subjects were Caucasian (n = 5, 55.6%).

### 3.3 Japanese cohort

All 5 Japanese DPN subjects had a diagnosis of Type 2 diabetes and were older than 41, with the average age around 63. Most subjects were male (n = 4, 80.0%).

Overall, 5 Japanese PHN subjects participated in the interviews. All but one of these subjects was above the age of 71 (n = 4, 80.0%) with the remaining subject in the age range of 61‐70 (n = 1, 20.0%) and 2 subjects were male (n = 2, 40.0%).

### 3.4 Patient‐reported symptoms

Table 2 summarizes the most frequent, spontaneously reported and probed neuropathic pain symptoms across both aetiologies and study populations. The most frequently reported symptom in DPN was numbness, and in PHN, the most frequently reported symptoms were burning and itchiness (US) and hypersensitivity and tingling (Japan). Location of pain was different between PHN and DPN subjects; most subjects with DPN experienced pain in their lower body extremity (eg feet and ankles) while PHN subjects experienced pain primarily in the chest and back areas.

**Table 2:** Most frequently reported symptoms in total population of US and Japanese subjects with DPN and PHN

Symptom	DPN		PHN	
	US (N = 10) N (%)	Japan (N = 5) N (%)	US (N = 9) N (%)	Japan (N = 5) N (%)
Ache/soreness	5 (50.0)	1 (20.0)	5 (55.6)	0 (0.0)
Burning	6 (60.0)	1 (20.0)	6 (66.7)	2 (40.0)
Hypersensitivity	2 (20.0)	2 (40.0)	5 (55.6)	2 (40.0)
Itchiness	2 (20.0)	0 (0.0)	6 (66.7)	0 (0.0)
Numbness	10 (100.0)	5 (100.0)	0 (0.0)	1 (20.0)
Tingling	9 (90.0)	2 (40.0)	1 (11.1)	2 (40.0)
Sharp pain	5 (50.0)	0 (0.0)	3 (33.3)	0 (0.0)

DPN, diabetic peripheral neuropathy; PHN, post‐herpetic neuralgia; US, United States.

### 3.5 US cohort

Numbness was reported by every US DPN subject (100.0%). Some subjects had difficulties characterizing their numbness beyond “numbness,” while other subjects described the sensation as “a loss of sense of feeling” or “doesn't have any sensation.” Subjects tended to describe numbness in regard to how frequently it occurred and most reported experiencing their numbness “constantly.” Tingling (90.0%) was also frequently reported and described using expressions such as “walking on rocks” and feeling like the foot “fell asleep.” All 9 subjects described their tingling by severity indicating that inactivity (typically in the evenings) led to feeling the tingling sensation more acutely. Subjects also frequently reported burning (60.0%), sharp pain (50.0%) and ache/soreness (50.0%). While the frequency of the burning sensation varied among subjects, most described the sensation as “intense” and “very painful.” Descriptions ranged from “burning sensation,” to “hot feet,” to “on fire,” and wanting to “put feet in ice water.” Most subjects described burning and tingling as concurrent or similar sensations. Descriptors of sharp pain varied among subjects from “crushing pain” to “splitting” and “shooting” to being “stuck with a pin” or “sharp needle.” However, all subjects described the sensation as sudden or spontaneous, frequent and short lasting. Ache/soreness was related to their level of physical activity or exertion, and relatively frequent and long in duration. However, common descriptors included a less severe sensation, such as “solid ache” or “dull ache.”

Among US PHN subjects, burning (66.7%), itchiness (66.7%), ache/soreness (55.6%) and hypersensitivity (55.6%) were the most frequently reported sensations. Most subjects described burning as a “feeling of heat” on the skin, as if someone had “thrown hot oil on you” and as frequent in occurrence but short in duration. Burning was also generally described as “intense” or “excruciating.” However, 2 subjects described their burning as a deeper, “more muscular” sensation, which was mild in severity but constantly present. In regard to itchiness, 3 subjects focused on the depth of the sensation describing it as “skin‐deep” or “internal.” In general, itchiness was not described as very severe. Frequency descriptions varied significantly between subjects and ranged from once a week to constantly. Ache/soreness was described by PHN subjects as if one had “hit your arm” or your body had been “beat on one side” with severity ranging from causing one to “scream” or “cry” to “a little sore.” In terms of frequency, subjects experienced aching/soreness as varying on a daily basis or as a constant sensation. Subjects described hypersensitivity in regard to their skin being “very sensitive to touch” and frequently reported having to avoid physical contact. Generally, subjects discussed hypersensitivity in regard to their outbreak period and not as a constant sensation.

### 3.6 Japanese cohort

All Japanese DPN subjects reported numbness (100.0%). Coldness, hypersensitivity and tingling were somewhat frequently reported (40.0%) by these subjects.

Among the Japanese PHN subjects, no more than 2 subjects reported experiencing the same symptom. However, hypersensitivity, shooting, throbbing and tingling were somewhat frequently reported (40.0%).

### 3.7 Saturation

Saturation was demonstrated on the total sample level with total number of concepts, and on an individual concept level with subjects grouped as units of analysis. Only spontaneously reported concepts were included in the saturation grid. One concept (cramping) emerged in the second to last subject in the US DPN population. Additionally, in the sample of Japanese subjects, 3 new concepts emerged from 2 subjects (allodynia, pressure/squeezing and stiffness). Saturation was achieved in the US PHN population with no new concepts emerging in the last 4 interviews. However, in the sample of Japanese subjects, 1 concept (numbness) emerged, introduced by the first Japanese subject. Overall, the qualitative data demonstrated saturation and sample size was deemed adequate where additional interviewers were unlikely to produce any new information relevant to neuropathic pain. Tables 3 and 4 summarize the saturation for all DPN and PHN concepts, respectively.

**Table 3:** Saturation of DPN concepts for US and Japanese subjects

Concepts^a^	First 30% of interviews vs next 40%	First 70% of interviews vs next 30%	Total	Saturation achieved for US^b^	100% of US interviews vs Japanese interviews	Total US + Japan	Saturation achieved for US + Japan
Numbness	3 vs 4	7 vs 3	10	Yes	10 vs 5	15	Yes
Tingling	3 vs 3	6 vs 3	9	Yes	9 vs 2	11	Yes
Burning	2 vs 2	4 vs 2	6	Yes	6 vs 0	6	Yes
Sharp	2 vs 2	4 vs 1	5	Yes	5 vs 0	5	Yes
Ache/soreness	0 vs 1	1 vs 2	3	Yes	3 vs 1	4	Yes
Cold	1 vs 1	2 vs 0	2	Yes	2 vs 2	4	Yes
Shooting	1 vs 2	3 vs 0	3	Yes	3 vs 1	4	Yes
Hypersensitivity	2 vs 0	2 vs 0	2	Yes	2 vs 1	3	Yes
Itchiness	1 vs 1	2 vs 0	2	Yes	2 vs 0	2	Yes
Pins and needles	0 vs 2	2 vs 0	2	Yes	2 vs 0	2	Yes
Allodynia	0 vs 0	0 vs 0	0	n/a	0 vs 1	1	Questionable
Cramping	0 vs 0	0 vs 1	1	Questionable	1 vs 0	1	Yes
Pressure/squeezing	0 vs 0	0 vs 0	0	n/a	0 vs 1	1	Questionable
Stiffness	0 vs 0	0 vs 0	0	n/a	0 vs 1	1	Questionable
Swelling	1 vs 0	1 vs 0	1	Yes	1 vs 0	1	Yes

DPN, diabetic peripheral neuropathy; n/a, not applicable; US, United States.

a Only spontaneously reported concepts are included in the saturation grid. Saturation analysis was conducted across the total sample with group sizes: n = 3 (group 1 [first 30%]), n = 4 (group 2 [second 40%]), n = 3 (group 3 [third 30%]).

b Concepts are considered “saturated” when no new concepts emerge in the last set of patient interviews.

**Table 4:** Saturation of PHN concepts for US and Japanese subjects

Concepts^a^	First 33.4% of interviews vs next 33.3%	First 66.6% of interviews vs next 33.3%	Total	Saturation achieved for US^b^	100% of US interviews vs Japanese interviews	Total US + Japan	Saturation achieved for US + Japan
Itchiness	2 vs 1	3 vs 2	5	Yes	5 vs 0	5	Yes
Ache/soreness	1 vs 2	3 vs 2	5	Yes	5 vs 0	5	Yes
Burning	3 vs 0	3 vs 1	4	Yes	4 vs 2	6	Yes
Hypersensitivity	2 vs 0	2 vs 2	4	Yes	4 vs 1	5	Yes
Stabbing	2 vs 0	2 vs 2	4	Yes	4 vs 1	5	Yes
Throbbing	1 vs 0	1 vs 1	2	Yes	2 vs 2	4	Yes
Sharp	0 vs 1	1 vs 2	3	Yes	3 vs 0	3	Yes
Shocking	0 vs 1	1 vs 2	3	Yes	3 vs 2	5	Yes
Tightness	1 vs 1	2 vs 0	2	Yes	2 vs 1	3	Yes
Tingling	1 vs 0	1 vs 0	1	Yes	1 vs 2	3	Yes
Piercing	1 vs 1	2 vs 0	2	Yes	2 vs 0	2	Yes
Stinging	1 vs 0	1 vs 1	2	Yes	2 vs 0	2	Yes
Gripping	1 vs 0	1 vs 0	1	Yes	1 vs 0	1	Yes
Numbness	0 vs 0	0 vs 0	0	n/a	0 vs 1	1	Questionable
Pinching	1 vs 0	1 vs 0	1	Yes	1 vs 0	1	Yes
Sticking	0 vs 1	1 vs 0	1	Yes	1 vs 0	1	Yes
Swelling	1 vs 0	1 vs 0	1	Yes	1 vs 0	1	Yes
Tension	1 vs 0	1 vs 0	1	Yes	1 vs 0	1	Yes
Warmth	0 vs 1	1 vs 0	1	Yes	1 vs 0	1	Yes

n/a, not applicable; PHN, post‐herpetic neuralgia; US, United States.

a Only spontaneously reported concepts are included in the saturation grid. Saturation analysis was conducted across the total sample with group sizes: n = 3 (group 1 [first 33.4%]), n = 3 (group 2 [second 33.3%]), n = 3 (group 3 [third 33.3%]).

b Concepts are considered “saturated” when no new concepts emerge in the last set of patient interviews.

### 3.8 Patient‐reported impacts

Table 5 summarizes the most frequent, spontaneously reported impacts across both aetiologies and study populations associated with neuropathic pain as described by interviewed subjects.

**Table 5:** Most frequently reported impacts for US and Japanese subjects with DPN and PHN

Impacts	DPN		PHN	
	US (N = 10) N (%)	Japan (N = 5) N (%)	US (N = 9) N (%)	Japan (N = 5) N (%)
Cognitive functioning				
Distracted by pain	7 (70.0)	0 (0.0)	0 (0.0)	3 (60.0)
Daily activities				
Clothing limitations	5 (50.0)	0 (0.0)	5 (55.6)	3 (60.0)
Household activities	5 (50.0)	0 (0.0)	6 (66.7)	4 (80.0)
Hygiene/personal care	0 (0.0)	3 (60.0)	6 (66.7)	0 (0.0)
Leisure activities	7 (70.0)	3 (60.0)	0 (0.0)	4 (80.0)
Emotional impact				
Angry/frustrated/aggravated	5 (50.0)	3 (60.0)	8 (88.9)	0 (0.0)
Anxious	0 (0.0)	4 (80.0)	0 (0.0)	0 (0.0)
Depressed/sad	5 (50.0)	0 (0.0)	6 (66.7)	3 (60.0)
Despair/hopelessness	0 (0.0)	3 (60.0)	0 (0.0)	0 (0.0)
Loss of motivation/interest	0 (0.0)	0 (0.0)	0 (0.0)	3 (60.0)
Social impact				
Fear/worried	5 (50.0)	3 (60.0)	0 (0.0)	0 (0.0)
Social activities	0 (0.0)	0 (0.0)	6 (66.7)	3 (60.0)
Relationship changes	0 (0.0)	0 (0.0)	6 (66.7)	0 (0.0)
Relying on others	5 (50.0)	0 (0.0)	0 (0.0)	3 (60.0)
Physical impact				
Avoid physical activities	0 (0.0)	0 (0.0)	0 (0.0)	4 (80.0)
Avoid physical contact	0 (0.0)	0 (0.0)	7 (77.8)	0 (0.0)
Body position	0 (0.0)	0 (0.0)	5 (55.6)	0 (0.0)
Difficulty falling asleep	0 (0.0)	3 (60.0)	0 (0.0)	0 (0.0)
Exercise/sports	0 (0.0)	0 (0.0)	0 (0.0)	3 (60.0)
Going outside	0 (0.0)	0 (0.0)	0 (0.0)	3 (60.0)
Lying to upright position	0 (0.0)	0 (0.0)	0 (0.0)	3 (60.0)
Sex life	0 (0.0)	0 (0.0)	5 (55.6)	0 (0.0)
Sleep disturbance	8 (80.0)	0 (0.0)	8 (88.9)	4 (80.0)
Standing	0 (0.0)	3 (60.0)	0 (0.0)	0 (0.0)
Up/down stairs	5 (50.0)	3 (60.0)	0 (0.0)	0 (0.0)
Walking	7 (70.0)	3 (60.0)	0 (0.0)	0 (0.0)

DPN, diabetic peripheral neuropathy; PHN, post‐herpetic neuralgia US, United States.

### 3.9 US cohort

Among the 10 US DPN subjects, 43 impacts were reported and divided into 10 domains: physical mobility (15 concepts), activities of daily living (7 concepts), emotional (7 concepts), sleep (3 concepts), social (3 concepts), cognitive functioning (2 concepts), physical (2 concepts), work/school (2 concepts), financial (1 concept) and leisure (1 concept).

Across all domains, the most frequently reported impact was sleep disturbance (80%), commonly attributed to waking up during the night due to pain and subsequently being unable to fall asleep. In the physical mobility domain, subjects frequently reported difficulty walking (70%) and difficulty going up/down stairs (50%) because of their pain; although for some subjects, it was because of their fear of falling, which was caused by numbness in their feet.

Among impacts on daily living activities, subjects frequently reported impact on ability to wear certain clothing (50%), specifically footwear, and difficulty completing household activities (50%), which subjects often attributed to physical mobility limitations (eg difficulty standing or kneeling).

Within the emotional domain, subjects frequently reported feeling angry/irritable (50%), depressed/sad (50%) and fear/worried (50%). Subjects describing feelings of anger/irritability noted that these feelings manifested as short temperedness and impatience around friends or family because of their pain. Most subjects described their fear or worry in terms of falling.

Within the social domain, the only frequently reported impact was relying on others (50%), which was closely linked with subjects’ difficulty completing household activities. Impact on subjects’ ability to partake in leisure activities related to physical mobility (70.0%). Within the cognitive functioning domain, subjects frequently reported instances of being distracted by pain (70.0%).

Among the US PHN subjects, 47 impacts were reported and divided into 10 domains: emotional (11 concepts), activities of daily living (10 concepts), physical mobility (9 concepts), social impacts (5 concepts), physical (4 concepts), sleep (3 concepts), leisure (2 concepts), cognitive functioning (1 concept), sexual (1 concept) and work/school (1 concept).

Overall, the most frequently reported impact was sleep disturbance (88.9%), primarily attributed to waking up due to pain. Two of the 11 reported emotional impacts were frequently reported by subjects: feeling angry/frustrated/aggravated (88.9%) and feeling depressed or sad (66.7%). Subjects who described feelings of anger/frustration/aggravation noted that these feelings manifested as short temperedness around friends or family.

Among frequently reported impacts of daily living, subjects frequently reported impact on their ability to complete household activities (66.7%), practice good hygiene and personal care (66.7%) and wear certain clothing (55.6%).

In the physical mobility domain, subjects frequently reported limitations on body position (55.6%), particularly reclining or laying on their back.

In regard to social impacts, subjects frequently reported their pain interfering with social activities (66.7%) and causing relationship changes with friends or family (66.7%). Subjects described relationship changes as linked to antisocial behaviour; with the underlying issue being feeling bothered by friends and family when in pain. Two of these subjects (22.2%) attributed the interference in social activities to this short temperedness and thus chose not to attend social events. In addition, 2 subjects (22.2%) were scared of potential pain that could occur while attending an event, and thus would forego certain events; 1 subject (11.1%) limited the length of time they could attend events. Reliance on others was commonly linked with subjects’ difficulty completing household activities.

The only frequently reported impact in the physical domain was avoiding physical contact to prevent triggering/exacerbating pain (77.8%) (ie touch from others, clothing or themselves). Lastly, subjects frequently reported that their pain experience interfered with their sex life (55.6%) attributing this impact more often to a loss of desire when in pain as opposed to a physical limitation. No frequently reported impacts were part of the cognitive functioning or work/school domains.

### 3.10 Japanese cohort

Among the 5 Japanese DPN subjects, 39 impacts were reported and divided into 10 domains: physical mobility (12 concepts), emotional (9 concepts), activities of daily living (4 concepts), social (4 concepts), work/school (3 concepts), physical (2 concepts), sleep (2 concepts), appearance (1 concept), cognitive functioning (1 concept) and leisure (1 concept).

Frequently reported impacts in the physical mobility domain were difficulty going up and down stairs (60.0%), standing (60.0%) and walking (60.0%). Of the emotional impacts, frequently reported concepts were feeling anxious (80.0%), feeling angry/frustrated/aggravated (60.0%), having a sense of despair/hopelessness (60.0%) and feeling fear/worry (60.0%).

Within the activities of daily living domain, the only frequently reported impact was hygiene/personal care (60.0%). There were no frequently reported impacts in the social domain or in the work/school domain.

Within the sleep domain, difficulty falling asleep (60.0%) was the only frequently reported impact. Lastly, an impact on subjects’ ability to do leisure activities (60.0%) was also frequently reported. There were no impacts on cognitive or work/school domains that were frequently/consistently reported.

Among the 5 Japanese PHN subjects, a total of 37 impacts were reported and divided into 8 domains: emotional impacts (11 concepts), activities of daily living (7 concepts), physical mobility impacts (6 concepts), social impacts (5 concepts), sleep (3 concepts), cognitive functioning (2 concepts), leisure activities (2 concepts) and work/school (1 concept).

Frequently reported impacts in the emotional domain were feeling depressed (60.0%) and a loss of motivation/interest (60.0%).

Within the activities of daily living domain, frequently reported impacts included avoiding physical activities (80.0%), household activities (80.0%), clothing limitations (60.0%), participating in exercise/sports (60.0%) and going outside (60.0%). Of the impacts on physical mobility, difficulty getting to an upright position from a lying position (60.0%) was frequently reported by subjects.

Frequently reported social impacts were subjects’ pain interfering with social activities in general (60.0%) and having to rely on others (60.0%). In regard to impacts on sleep, sleep disturbance (80.0%) was reported by most subjects.

In regard to cognitive functioning, subjects frequently reported being distracted by their pain (60.0%). Lastly, an impact on subjects’ ability to partake in leisure activities (80.0%) was also frequently reported.

### 3.11 The conceptual model

**Figure 1:**
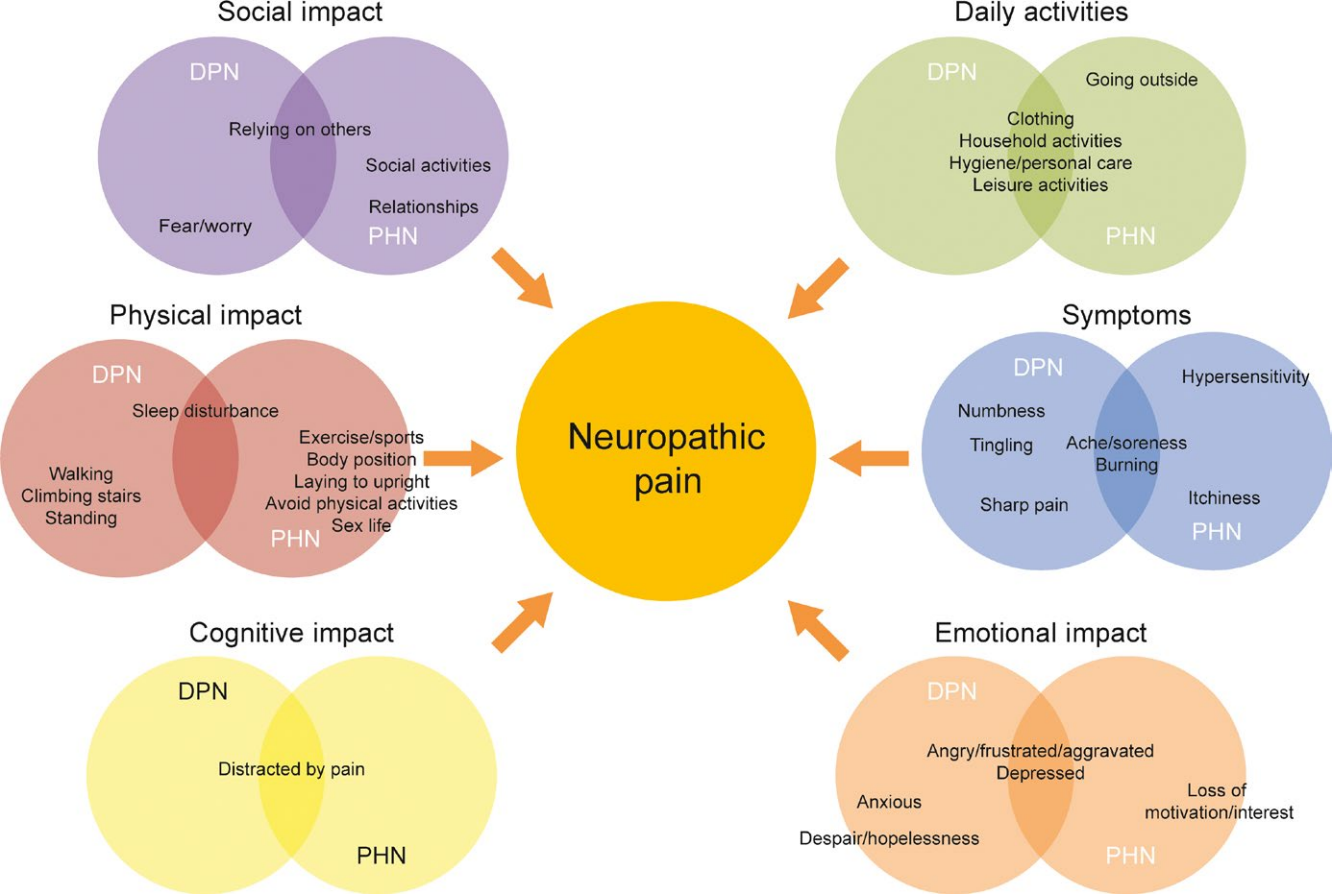
Conceptual model in DPN and PHN. DPN, diabetic peripheral neuropathy, PHN, post‐herpetic neuralgia

The conceptual model, shown in Figure 1, shows relevant concepts when they co‐occur (or do not) across DPN and PHN cohorts, with the colour of the circles indicating a different domain. Overall, concepts were found to be similar among both geographic populations; therefore, this model does not distinguish between Japan and the United States. Rather the focus of the model is on similarities and differences within the aetiologies, DPN and PHN. Frequency of reporting in this conceptual model is calculated by the number of subjects who reported a concept out of the total number of subjects in that geographical cohort (United States and Japan). In order for concepts to be included in the model, they only had to have been frequently reported in one of the geographical cohorts. Differences and similarities in symptom presentation between aetiologies are also expressed in this model by their location: in the largest 3 bubbles representing the most frequently reported symptoms, the location of the concepts shows where they skew in regard to each aetiology, that is numbness, tingling and sharp pain are the most prevalent in DPN while hypersensitivity and itchiness are more prevalent in PHN.

## 4 DISCUSSION

Few neuropathic pain studies have taken a patient‐centred approach in estimating symptom‐ and neuropathic pain‐specific impact burden. Crawford et al^21^ previously conducted research of a similar nature which was broader in scope (including data from the United States, Japan, Brazil, China, Finland and Spain), but utilized focus groups and only examined symptoms and not impacts of neuropathic pain, to evaluate the relevancy of a neuropathic pain‐specific measure. In addition, their research was conducted prior to the FDA Guidance for Industry in PRO measures,^24^ which tends to favour individual interviews that allow spontaneous reporting of concepts (as opposed to 1 participant introducing the concept for everyone in a focus group) and analysis of the data under the principle of saturation.

Although there are common symptoms across DPN and PHN, selected symptoms should be considered specific to each aetiology. This research found that burning sensation and ache/soreness were common to both aetiologies, whereas numbness, tingling and sharp pain were more commonly reported in DPN, and itchiness and hypersensitivity were more commonly reported in PHN. Therefore, outcome measures should be specific to the underlying disease‐causing neuropathy or should focus on common core neuropathic pain symptoms with aetiology‐specific questions as a supplement.

Current thinking in the field of outcomes assessment suggests that *symptoms* are best collected on a daily basis in a diary.^25^ There are 2 reasons for this: to minimize recall bias that occurs with longer recall periods and to ensure that a complete picture of symptoms is collected when symptoms may vary day‐to‐day. Further, it has been suggested that daily recall of symptoms is best done by collecting the worst score for a symptom during a 24‐hour recall period, thereby focusing on the worst or most salient symptom experience in a given timeframe. This approach will likely be appropriate in DPN and PHN; however, some of the symptoms reported by subjects in this research may be more difficult to characterize on a full response scale from none to severe. For instance, numbness and ache/soreness may describe the symptom state in its entirety, that is there are no degrees of numbness or soreness, only presence or absence.

Overall, concepts in Japan were not qualitatively different from in the United States, but Japanese subjects did report fewer symptoms than US subjects. Even with probing specific concepts following the interview guide, Japanese subjects did not find the concepts relevant to their pain experience. A possible explanation could be that Japanese subjects do not talk about their pain as often or as much compared to US subjects (possibly due to the very limited time spent with clinicians at a given visit).

In terms of impacts, sleep problems were most frequently reported. For purely research purposes, there are several available PRO measures that may be used to assess sleep problems.^26^, ^27^, ^28^ However, for labelling and promotional claims in the area of sleep problems, the recent FDA Guidance on analgesic trials^29^ discourages sponsors from using PRO measures while noting the requirement of objective sleep assessments.

In terms of physical impacts, problems with mobility were frequently reported. Difficulty walking and going up‐ and downstairs due to pain were the main issues; however, some subjects reported that numbness could throw them off balance or cause them to worry about falling. Issues with mobility can be collected in a variety of generic measures (eg Short Form [SF]‐36). In general, it appeared that PHN subjects had more physical mobility impacts than DPN subjects. Researchers may also want to consider more objective tests such as a 6‐minute walk test, the Timed Up and Go (TUG) test or a test of standing balance, all of which have been shown to be reliable assessments in peripheral neuropathy.^30^ Of the 3 tests, the TUG and standing balance tests may be more appropriate objective measures as they directly incorporate balance in the assessment. This research did not specifically deal with mobility issues in neuropathic pain in a great deal of depth. Further research should be conducted to the extent of mobility limitations before selecting a measurement strategy for clinical trials that is intended to lead to labelling and promotional claims.

Emotional and social impacts reported by subjects in this study were similar to impacts reported elsewhere in the literature.^13^, ^14^ Main impacts included feeling angry and worry, relying on others and partaking in leisure activities and other social activities. There are currently measures that exist that adequately cover these areas for research purposes and routine clinical monitoring. In terms of measures that could support labelling and promotional claims, it is unlikely that any existing measure could support claims in these areas and, furthermore, it is unlikely that newly developed measures that used a rigorous development and validation process could support these kinds of claims. The current thinking at the FDA would treat these concepts as distal to the core disease symptoms and therefore not likely candidates for claim.^31^

Some impacts were notably different between the Japanese vs US populations. In emotional impacts, the concept of despair/hopelessness was unique to the Japanese population. In the activities of daily living domain, 60% of DPN subjects in Japan reported a negative impact on hygiene, while no US DPN subjects reported the impact. Impact on driving/riding in a car was also lower in Japanese subjects, which may be explained by the density of Japanese urban areas and lower car ownership. In terms of social impacts, while PHN subjects in the United States reported a negative impact on sex life, no subjects in Japan (DPN or PHN) reported experiencing such impact.

Generic measures have been used frequently in neuropathic pain trials. Although the use of generic measures may be useful for getting a broad indication of the burden of neuropathic pain^13^, ^32^ and allow for the comparison of scores across disease areas (and to a reference group of normed subjects as is the case with the SF‐36), there is a need for more sensitive and responsive tools to track patient progress in areas that patients identify as important in routine practice and for evaluation in clinical trials.

The inadequacy of generic measures to capture treatment benefits in neuropathic pain was illustrated in the Vinik et al^33^ evaluation of 11 randomized, double‐blind trials of pregabalin for the treatment of DPN and PHN. Although the SF‐36 bodily pain domain showed significant decreases in pain in the pregabalin group compared to placebo, a mix of significant and non‐significant results were observed in role emotional, vitality and social functioning domains and non‐significant results were found in physical functioning and role‐physical domains. It is a matter of conjecture at this stage, but it is possible a more patient‐centred and neuropathic pain focused evaluation would have found different results.

Smith et al^34^ previously identified 3 good candidate disease‐specific HRQoL measures for use in clinical trials with adequate psychometric properties; however, all 3 measures were developed prior to both the draft and final FDA PRO Guidance and it is unclear to what extent they would be able to support an object of labelling or promotional claims in the United States.

## 5 CONCLUSIONS

A conceptual model explicitly outlines the discrete, relevant qualities of a phenomenon of interest (in this case, neuropathic pain). The FDA's 2009 PRO Guidance further elaborates that an “instrument can be used to measure the effect of a medical intervention on one or more *concepts* (ie the thing being measured, such as a symptom or group of symptoms, effects on a particular function or group of functions, or a group of symptoms or functions shown to measure the severity of a health condition).” As such, concepts refer to the *what* of a measurement with the conceptual model graphic helping to visualize at a single glance what the relevant concepts are as well as elucidating *how* the concepts, subconcepts and domains are related. The current conceptual model based on primary research with individuals with DPN or PHN identifies and summarizes what to measure from the patient's perspective.

Studying the conceptual model, several notable differences were observed from these interviews. Patients with DPN experienced pain sensation in the form of numbness and tingling in their lower body extremity, which coincide with physical impacts such as difficulty walking and standing. In comparison, pain in patients with PHN was primarily described in terms of burning and hypersensitivity, and its location was limited to their herpetic breakout areas; similar to the DPN subjects, their impact on daily living was directly related to their symptoms such as avoiding physical touch in their painful areas. The symptoms and impacts experienced by patients were similar in the United States and Japan. Based on the data from this research, researchers can begin to appropriately identify existing measures, determine if modifications are needed or develop new PRO measures that provide a sensitive and responsive method for evaluating treatment outcomes in neuropathic pain.

## AUTHORS’ CONTRIBUTIONS

SH, CE, BC, ME and FvN were involved in the design of the study and interpretation of results. SH, AR and TW were the primary interviewers and data analysts. All authors assisted in the drafting of the manuscript.

## CONFLICT OF INTEREST

SH, TW, AR and CE are employed by Endpoint Outcomes, FVN is employed by Dompé, and BC is employed by inVentiv Health. At the time of the study and during preparation of the manuscript, FvN was employed by Astellas and BC was employed by IMS Japan. ME is employed by Astellas. Endpoint Outcomes and IMS Japan received consulting fees from Astellas to conduct this work. There are no other competing interests.

## References

[1] Treede RD , Jensen TS , Campbell JN , et al. Neuropathic pain: redefinition and a grading system for clinical and research purposes. Neurology. 2008;70:1630‐1635. 18003941 10.1212/01.wnl.0000282763.29778.59

[2] Jensen MP , Chodroff MJ , Dworkin RH . The impact of neuropathic pain on health‐related quality of life: review and implications. Neurology. 2007;68:1178‐1182. 17420400 10.1212/01.wnl.0000259085.61898.9e

[3] Baron R , Binder A , Wasner G . Neuropathic pain: diagnosis, pathophysiological mechanisms, and treatment. Lancet Neurol. 2010;9:807‐819. 20650402 10.1016/S1474-4422(10)70143-5

[4] Stacey BR . Management of peripheral neuropathic pain. Am J Phys Med Rehabil. 2005;84:S4‐S16. 15722782

[5] van Hecke O , Austin SK , Khan RA , Smith BH , Torrance N . Neuropathic pain in the general population: a systematic review of epidemiological studies. Pain. 2014;155:654‐662. 24291734 10.1016/j.pain.2013.11.013

[6] Madhuvrata P , Cody JD , Ellis G , Herbison GP , Hay‐Smith EJ . Which anticholinergic drug for overactive bladder symptoms in adults. Cochrane Database Syst Rev. 2012;1:Cd005429. 22258963 10.1002/14651858.CD005429.pub2 PMC12989262

[7] Schmader KE . Epidemiology and impact on quality of life of postherpetic neuralgia and painful diabetic neuropathy. Clin J Pain. 2002;18:350‐354. 12441828 10.1097/00002508-200211000-00002

[8] Dieleman JP , Kerklaan J , Huygen FJ , Bouma PA , Sturkenboom MC . Incidence rates and treatment of neuropathic pain conditions in the general population. Pain. 2008;137:681‐688. 18439759 10.1016/j.pain.2008.03.002

[9] O'Connor AB , Dworkin RH . Treatment of neuropathic pain: an overview of recent guidelines. Am J Med. 2009;122:S22‐S32. 10.1016/j.amjmed.2009.04.007 19801049

[10] Gore M , Brandenburg NA , Hoffman DL , Tai KS , Stacey B . Burden of illness in painful diabetic peripheral neuropathy: the patients’ perspectives. J Pain. 2006;7:892‐900. 17157775 10.1016/j.jpain.2006.04.013

[11] Serpell M , Gater A , Carroll S , Abetz‐Webb L , Mannan A , Johnson R . Burden of post‐herpetic neuralgia in a sample of UK residents aged 50 years or older: findings from the Zoster Quality of Life (ZQOL) study. Health Qual Life Outcomes. 2014;12:92. 24920439 10.1186/1477-7525-12-92 PMC4063222

[12] O'Connor AB . Neuropathic pain: quality‐of‐life impact, costs and cost effectiveness of therapy. Pharmacoeconomics. 2009;27:95‐112. 19254044 10.2165/00019053-200927020-00002

[13] Langley PC , Van Litsenburg C , Cappelleri JC , Carroll D . The burden associated with neuropathic pain in Western Europe. J Med Econ. 2013;16:85‐95. 22970839 10.3111/13696998.2012.729548

[14] Alleman CJ , Westerhout KY , Hensen M , et al. Humanistic and economic burden of painful diabetic peripheral neuropathy in Europe: a review of the literature. Diabetes Res Clin Pract. 2015;109:215‐225. 26008721 10.1016/j.diabres.2015.04.031

[15] Closs SJ , Staples V , Reid I , Bennett MI , Briggs M . Managing the symptoms of neuropathic pain: an exploration of patients’ experiences. J Pain Symptom Manage. 2007;34:422‐433. 17583468 10.1016/j.jpainsymman.2006.12.004

[16] Attal N , Lanteri‐Minet M , Laurent B , Fermanian J , Bouhassira D . The specific disease burden of neuropathic pain: results of a French nationwide survey. Pain. 2011;152:2836‐2843. 22019149 10.1016/j.pain.2011.09.014

[17] de Andres J , de la Calle JL , Perez M , Lopez V . Clinical characteristics, patient‐reported outcomes, and previous therapeutic management of patients with uncontrolled neuropathic pain referred to pain clinics. Pain Res Treat. 2014;2014:518716. 24891950 10.1155/2014/518716 PMC4027022

[18] Doth AH , Hansson PT , Jensen MP , Taylor RS . The burden of neuropathic pain: a systematic review and meta‐analysis of health utilities. Pain. 2010;149:338‐344. 20227832 10.1016/j.pain.2010.02.034

[19] Schaefer C , Mann R , Sadosky A , et al. Burden of illness associated with peripheral and central neuropathic pain among adults seeking treatment in the United States: a patient‐centered evaluation. Pain Med. 2014;15:2105‐2119. 25039856 10.1111/pme.12502 PMC4491355

[20] Lowe MM , Blaser DA , Cone L , et al. Increasing patient involvement in drug development. Value Health. 2016;19:869‐878. 27712716 10.1016/j.jval.2016.04.009

[21] Crawford B , Bouhassira D , Wong A , Dukes E . Conceptual adequacy of the neuropathic pain symptom inventory in six countries. Health Qual Life Outcomes. 2008;6:62. 18706109 10.1186/1477-7525-6-62 PMC2531087

[22] Glaser BG , Strauss AL . The Discovery of Grounded Theory: Strategies for Qualitative Research. New York, NY: Aldine de Gruyter; 1967.

[23] Charmaz K . Grounded Theory. London: Sage Publications; 1995.

[24] U.S. Department of Health and Human Services Food and Drug Administration (FDA) , Center for Drug Evaluation and Research (CDER) , Center for Biologics Evaluation and Research (CBER) , and Center for Devices and Radiological Health (CDRH) . Guidance for industry. Patient‐reported outcome measures: use in medical product development to support labeling claims; 2009.

[25] Stull DE , Leidy NK , Parasuraman B , Chassany O . Optimal recall periods for patient‐reported outcomes: challenges and potential solutions. Curr Med Res Opin. 2009;25:929‐942. 19257798 10.1185/03007990902774765

[26] Rand Health . MOS Sleep Scale Survey Instrument. https://www.rand.org/content/dam/rand/www/external/health/surveys_tools/mos/mos_sleep_survey.pdf. Accessed on December 1 2017

[27] Parrott AC , Hindmarch I . The Leeds Sleep Evaluation Questionnaire in psychopharmacological investigations – a review. Psychopharmacology. 1980;71:173‐179. 6777817 10.1007/BF00434408

[28] Buysse DJ , Reynolds 3rd CF , Monk TH , Berman SR , Kupfer DJ . The Pittsburgh Sleep Quality Index: a new instrument for psychiatric practice and research. Psychiatry Res. 1989;28:193‐213. 2748771 10.1016/0165-1781(89)90047-4

[29] U.S. Department of Health and Human Services Food and Drug Administration (FDA) , Center for Drug Evaluation and Research (CDER) , Center for Biologics Evaluation and Research (CBER) , and Center for Devices and Radiological Health (CDRH) . Guidance for industry. Analgesic indications: developing drug and biological products draft guidance; 2014.

[30] Manor B , Doherty A , Li L . The reliability of physical performance measures in peripheral neuropathy. Gait Posture. 2008;28:343‐346. 18321706 10.1016/j.gaitpost.2008.01.004

[31] Pecha S , Kennergren C , Yildirim Y , et al. Coronary sinus lead removal: a comparison between active and passive fixation leads. PLoS ONE. 2016;11:e0153651. 27119368 10.1371/journal.pone.0153651 PMC4847909

[32] Dermanovic Dobrota V , Hrabac P , Skegro D , et al. The impact of neuropathic pain and other comorbidities on the quality of life in patients with diabetes. Health Qual Life Outcomes. 2014;12:171. 25468384 10.1186/s12955-014-0171-7 PMC4264315

[33] Vinik A , Emir B , Cheung R , Whalen E . Relationship between pain relief and improvements in patient function/quality of life in patients with painful diabetic peripheral neuropathy or postherpetic neuralgia treated with pregabalin. Clin Ther. 2013;35:612‐623. 23541708 10.1016/j.clinthera.2013.03.008

[34] Smith SC , Lamping DL , MacLaine GD . Measuring health‐related quality of life in diabetic peripheral neuropathy: a systematic review. Diabetes Res Clin Pract. 2012;96:261‐270. 22154463 10.1016/j.diabres.2011.11.013

