# Supplementary material for: Neuropathic pain: A patient‐centred approach to measuring outcomes
Source: Health Expect. 2018 Apr 15;21(4):774–86. doi: 10.1111/hex.12673 (PMC6117483; doi:10.1111/hex.12673)
Supplement: Supplementary file 1 [file HEX-21-774-s001.docx]

Neuropathic Pain Symptoms

[Duration: 30/60 minutes]

Objective:

To gather spontaneous information about the patient’s treatment related symptoms.

***Note to Interviewer****: Active listening is key to an interview; actively listen for and write down key concepts elicited by patients in their own words.*

1. How do you refer to your condition? What’s the word that you use to refer to your condition? Are you familiar with the term: *diabetic peripheral neuropathy / post-herpetic neuralgia*

*Listen for:*

- *Their spontaneous mention of “neuropathic pain”*

*Probe if not mentioned:*

- Have you heard of the term “neuropathic pain”?

1. When did you first see a health professional in relation to your *diabetic peripheral neuropathy / post-herpetic neuralgia*
   1. Okay, so that was [*x years/months ago*]. Who did you see?
   2. What prompted you to see them? What were you experiencing?
   3. Who else have you seen since then? Have you talked to anyone else about your condition? If so, who? If not, why?
2. What symptoms do you experience currently? How would you describe the [*symptom*] for me?
   1. What does it feel like?

*If difficulty responding, ask in a different way such as: “For instance, how would you describe what the symptom is like to a friend or someone who does not have this kind of pain?”*

*Listen for (not to be probed unless patient has extreme difficulty describing their symptoms and until adequate time has been given to the subject to respond on their own):*

- *Sensations such as burning or ice cold (numbness) or like “pins and needles”*
- *Pain that is sudden, described as shooting, stabbing, or like electric shocks*
- *Pain that is spontaneous (i.e., arising without stimulus)*
- *An exaggerated response to something that causes pain (hyperalgesia)*
- *Pain caused by something that normally would not cause pain (allodynia)*
- *Itching sensation*
- *Negative symptoms such as weakness or changes in reflexes*
- *For cLBP only: Listen for pain described as dull, aching, or throbbing pain.*
  1. Where do you experience the [*symptom*]? Do they feel different based on the location?
  2. How is the sensation different than other kind of pain you’ve experienced in the past? Do you have any other conditions that cause you chronic pain?
  3. How often do you experience this [*symptom*]? (e.g., every day? every other day? 2-3 times a week? 2-3 times a month?)
  4. How long does the [*symptom*] typically last? (e.g., minutes? hours? days?)
  5. How are your symptoms triggered? Do you notice any patterns or do they happen randomly without a trigger?
  6. Have your symptoms changed since your diagnosis? If yes, then how did they change?

*Listen for:*

- *Severity change [e.g., “the pain has gotten worse”]*
- *Frequency change [e.g., “I experience the pain more often now or lasts longer”]*
- *Description change [e.g., “the pain feels different than before”])*
  1. How do your symptoms change from day to day or within a day (i.e., change throughout the day from day into night)? How do they change (if at all) from week to week or depending on time of the year?

*Listen for:*

- *Depending on weather (e.g., from warmer months to colder, if applicable)?*
- *Aforementioned severity, frequency or description change depending on time of day)*
  1. On a scale of one to 10 (1=“No [*symptom*]” and 10=“[*Symptom*] as bad you can imagine”), how would you rate your [*symptom*] on a typical day? On a bad day? On a good day?

*Probe:*

- *What’s a “good” day like with your [PHN or DPN or cLBP]? What makes it a good day?*
- *What’s a “bad” like? What makes it a bad day?*

| Location | Experience (✓) | Description (e.g., adjectives used) | Severity/Frequency/Duration |
| --- | --- | --- | --- |
| Arms |  |  |  |
| Fingers |  |  |  |
| Toes |  |  |  |
| Legs |  |  |  |
| Back |  |  |  |
| Head |  |  |  |
| Neck |  |  |  |
| Shoulder |  |  |  |
| Other [specify]  ______________ |  |  |  |
| Other [specify]  ______________ |  |  |  |
| Other [specify]  ______________ |  |  |  |

***Worst Pain***

1. What were the **last 24 hours** like in terms of your neuropathic pain? If I ask you, “*what was the* ***worst neuropathic pain*** *you have experienced* ***in the last 24 hours*** *like*?”, how would you respond?
2. Can you describe what this pain felt like?
3. How long did this pain last?
4. **How intense** did your pain get at its worst?
   1. On a scale of 0 to 10, where 0 is no [symptom] and 10 [symptom] as intense as you can imagine, how intense does it get?
5. How often does the worst pain occur?

***Bothersomeness of Symptoms***

- For the next part of the interview, I’d like you to tell me overall how bothersome each symptom is to you. A “0” means not bothersome at all and a “10” means extremely bothersome.
- When you hear the word bothersome, what does it mean? How are the symptoms bothersome?

| **Note**: Use the worksheet on the next page to list each symptom the subject mentioned during the interview and ask them to rate how bothersome each symptom is to them. |
| --- |

**BOTHERSOME RATINGS - SYMPTOMS**

**Instructions:** Please rate how bothersome **overall** each neuropathic pain symptom is to you.

1. Overall, how bothersome to you is ____________ ?

| Not bothersome  at all | | | | |  | |  | | | | | | | | | | |  | | | Extremely  bothersome | | | | | |
| --- | --- | --- | --- | --- | --- | --- | --- | --- | --- | --- | --- | --- | --- | --- | --- | --- | --- | --- | --- | --- | --- | --- | --- | --- | --- | --- |
| ▼ | | | | |  | |  | | | | | | | | | | |  | | | ▼ | | | | | |
|  | 0 | | 1 | | 2 | | | 3 | | 4 | | 5 | | 6 | | 7 | | | 8 | | | 9 | | 10 | |  |
|  |  |  |  |  |  |  | |  |  |  |  |  |  |  |  |  |  | |  |  | |  |  |  |  |  |
|  |  | |  | |  | | |  | |  | |  | |  | |  | | |  | | |  | |  | |  |

Overall, how bothersome to you is ____________ ?

| Not bothersome  at all | | | | |  | |  | | | | | | | | | | |  | | | Extremely  bothersome | | | | | |
| --- | --- | --- | --- | --- | --- | --- | --- | --- | --- | --- | --- | --- | --- | --- | --- | --- | --- | --- | --- | --- | --- | --- | --- | --- | --- | --- |
| ▼ | | | | |  | |  | | | | | | | | | | |  | | | ▼ | | | | | |
|  | 0 | | 1 | | 2 | | | 3 | | 4 | | 5 | | 6 | | 7 | | | 8 | | | 9 | | 10 | |  |
|  |  |  |  |  |  |  | |  |  |  |  |  |  |  |  |  |  | |  |  | |  |  |  |  |  |
|  |  | |  | |  | | |  | |  | |  | |  | |  | | |  | | |  | |  | |  |

Overall, how bothersome to you is ____________ ?

| Not bothersome  at all | | | | |  | |  | | | | | | | | | | |  | | | Extremely  bothersome | | | | | |
| --- | --- | --- | --- | --- | --- | --- | --- | --- | --- | --- | --- | --- | --- | --- | --- | --- | --- | --- | --- | --- | --- | --- | --- | --- | --- | --- |
| ▼ | | | | |  | |  | | | | | | | | | | |  | | | ▼ | | | | | |
|  | 0 | | 1 | | 2 | | | 3 | | 4 | | 5 | | 6 | | 7 | | | 8 | | | 9 | | 10 | |  |
|  |  |  |  |  |  |  | |  |  |  |  |  |  |  |  |  |  | |  |  | |  |  |  |  |  |
|  |  | |  | |  | | |  | |  | |  | |  | |  | | |  | | |  | |  | |  |

Overall, how bothersome to you is ____________ ?

| Not bothersome  at all | | | | |  | |  | | | | | | | | | | |  | | | Extremely  bothersome | | | | | |
| --- | --- | --- | --- | --- | --- | --- | --- | --- | --- | --- | --- | --- | --- | --- | --- | --- | --- | --- | --- | --- | --- | --- | --- | --- | --- | --- |
| ▼ | | | | |  | |  | | | | | | | | | | |  | | | ▼ | | | | | |
|  | 0 | | 1 | | 2 | | | 3 | | 4 | | 5 | | 6 | | 7 | | | 8 | | | 9 | | 10 | |  |
|  |  |  |  |  |  |  | |  |  |  |  |  |  |  |  |  |  | |  |  | |  |  |  |  |  |
|  |  | |  | |  | | |  | |  | |  | |  | |  | | |  | | |  | |  | |  |

Overall, how bothersome to you is ____________ ?

| Not bothersome  at all | | | | |  | |  | | | | | | | | | | |  | | | Extremely  bothersome | | | | | |
| --- | --- | --- | --- | --- | --- | --- | --- | --- | --- | --- | --- | --- | --- | --- | --- | --- | --- | --- | --- | --- | --- | --- | --- | --- | --- | --- |
| ▼ | | | | |  | |  | | | | | | | | | | |  | | | ▼ | | | | | |
|  | 0 | | 1 | | 2 | | | 3 | | 4 | | 5 | | 6 | | 7 | | | 8 | | | 9 | | 10 | |  |
|  |  |  |  |  |  |  | |  |  |  |  |  |  |  |  |  |  | |  |  | |  |  |  |  |  |
|  |  | |  | |  | | |  | |  | |  | |  | |  | | |  | | |  | |  | |  |

Overall, how bothersome to you is ____________ ?

| Not bothersome  at all | | | | |  | |  | | | | | | | | | | |  | | | Extremely  bothersome | | | | | |
| --- | --- | --- | --- | --- | --- | --- | --- | --- | --- | --- | --- | --- | --- | --- | --- | --- | --- | --- | --- | --- | --- | --- | --- | --- | --- | --- |
| ▼ | | | | |  | |  | | | | | | | | | | |  | | | ▼ | | | | | |
|  | 0 | | 1 | | 2 | | | 3 | | 4 | | 5 | | 6 | | 7 | | | 8 | | | 9 | | 10 | |  |
|  |  |  |  |  |  |  | |  |  |  |  |  |  |  |  |  |  | |  |  | |  |  |  |  |  |
|  |  | |  | |  | | |  | |  | |  | |  | |  | | |  | | |  | |  | |  |

Overall, how bothersome to you is ____________ ?

| Not bothersome  at all | | | | |  | |  | | | | | | | | | | |  | | | Extremely  bothersome | | | | | |
| --- | --- | --- | --- | --- | --- | --- | --- | --- | --- | --- | --- | --- | --- | --- | --- | --- | --- | --- | --- | --- | --- | --- | --- | --- | --- | --- |
| ▼ | | | | |  | |  | | | | | | | | | | |  | | | ▼ | | | | | |
|  | 0 | | 1 | | 2 | | | 3 | | 4 | | 5 | | 6 | | 7 | | | 8 | | | 9 | | 10 | |  |
|  |  |  |  |  |  |  | |  |  |  |  |  |  |  |  |  |  | |  |  | |  |  |  |  |  |
|  |  | |  | |  | | |  | |  | |  | |  | |  | | |  | | |  | |  | |  |

Overall, how bothersome to you is ____________ ?

| Not bothersome  at all | | | | |  | |  | | | | | | | | | | |  | | | Extremely  bothersome | | | | | |
| --- | --- | --- | --- | --- | --- | --- | --- | --- | --- | --- | --- | --- | --- | --- | --- | --- | --- | --- | --- | --- | --- | --- | --- | --- | --- | --- |
| ▼ | | | | |  | |  | | | | | | | | | | |  | | | ▼ | | | | | |
|  | 0 | | 1 | | 2 | | | 3 | | 4 | | 5 | | 6 | | 7 | | | 8 | | | 9 | | 10 | |  |
|  |  |  |  |  |  |  | |  |  |  |  |  |  |  |  |  |  | |  |  | |  |  |  |  |  |
|  |  | |  | |  | | |  | |  | |  | |  | |  | | |  | | |  | |  | |  |

Overall, how bothersome to you is ____________ ?

| Not bothersome  at all | | | | |  | |  | | | | | | | | | | |  | | | Extremely  bothersome | | | | | |
| --- | --- | --- | --- | --- | --- | --- | --- | --- | --- | --- | --- | --- | --- | --- | --- | --- | --- | --- | --- | --- | --- | --- | --- | --- | --- | --- |
| ▼ | | | | |  | |  | | | | | | | | | | |  | | | ▼ | | | | | |
|  | 0 | | 1 | | 2 | | | 3 | | 4 | | 5 | | 6 | | 7 | | | 8 | | | 9 | | 10 | |  |
|  |  |  |  |  |  |  | |  |  |  |  |  |  |  |  |  |  | |  |  | |  |  |  |  |  |
|  |  | |  | |  | | |  | |  | |  | |  | |  | | |  | | |  | |  | |  |

Neuropathic Pain Impact

[Duration: 25/60 minutes]

| ***Diagnosis, symptoms, and impact*** |
| --- |

**Note:** Use the impact tracking grid at the end of this interview guide to track the impacts of neuropathic pain discussed by subjects.

| **Note:** For each impact, ask the following questions: | |
| --- | --- |
| - Were there **specific symptoms** that caused [impact]? If so, which symptoms? - **When** do you experience [impact]? - **How often** do you experience [impact]? - Do you think it is related to your [subject’s term for neuropathic pain]? Why or why not? - **How bad** is [impact] when you experience it? - [When appropriate for impact] **How long** does [impact] last when you experience it? - How does the [impact] make them feel? |  |

1. Earlier you mentioned [impact], could you please tell me a little more about that?
   1. How would you describe [impact] to a friend?

**Note:** After discussing impacts spontaneously described by subjects during Part 1, ask the following questions:

1. How does [subject’s term for neuropathic pain] **affect your life**? How different was your life before the disease developed?
   1. Does your [subject’s term for neuropathic pain] **change the way you do things**? If so, how?
      1. Does it **stop you from doing things**? What things? Why?
   2. Does your [subject’s term for neuropathic pain] affect your **daily activities**? If so, what activities?
   3. Does your [subject’s term for neuropathic pain] affect your **leisure activities** (i.e., what you like to do in your free time)? If so, how?
   4. Does your [subject’s term for neuropathic pain] affect your **social life**? If so, how?
   5. Does your [subject’s term for neuropathic pain] affect you at **work or school**? If so, how?
   6. Does your [subject’s term for neuropathic pain] affect you **emotionally**? If so, how?
   7. Are there any other impacts related to your [subject’s term for neuropathic pain] that you would like to discuss? **(If yes, ask questions about each impact as noted above)**

**Note**: Probe for the following impacts **ONLY** if they have not been discussed at this point. If the subject experiences an impact, ask questions noted above.

1. Have you ever experienced any of the following impacts that you feel are related to your [subject’s term for neuropathic pain]?

*Emotional or psychological*

- - - 1. How does it make you feel to be dealing with neuropathic pain? Has it changed the way you see yourself because of the disease? If so, how has it changed?
      2. Depressed
      3. Anxious
      4. Angry
      5. Frustrated
      6. Embarrassed
      7. Self-conscious

*Physical*

- - - 1. Mobility or physical ability to do things
      2. Interfere with sex life
      3. Sleep (Clarify: trouble falling asleep or staying asleep; Stiffness or pain in the morning)

*Mental*

- - - 1. Ability to concentrate or focus on things
      2. Memory
      3. Other mental function

*Impact on work or school*

- - - 1. Miss work or school
      2. Productivity at work or school
      3. Relationship with colleagues or boss

*Social*

- - - 1. Interfere with relationships
      2. Relying on others
      3. Interfere with social activities (i.e., staying home and/or changing/canceling plans)

*Impact on wearing clothes*

- - - 1. Avoid certain material of clothing (e.g., polyester) you want to wear due to allodynia or hyperalgesia

***Bothersomeness of Impacts***

- For the next part of the interview, I’d like you to tell me overall how bothersome each impact is to you. A “0” means not bothersome at all and a “10” means extremely bothersome.

| **Note**: Use the worksheet on the next page to list each symptom the subject mentioned during the interview and ask them to rate how bothersome each symptom is to them. |
| --- |

**BOTHERSOME RATINGS - IMPACTS**

**Instructions:** Please rate how bothersome **overall** each impact of neuropathic pain is to you.

1. Overall, how bothersome to you is ____________ ?

| Not bothersome  at all | | | | |  | |  | | | | | | | | | | |  | | | Extremely  bothersome | | | | | |
| --- | --- | --- | --- | --- | --- | --- | --- | --- | --- | --- | --- | --- | --- | --- | --- | --- | --- | --- | --- | --- | --- | --- | --- | --- | --- | --- |
| ▼ | | | | |  | |  | | | | | | | | | | |  | | | ▼ | | | | | |
|  | 0 | | 1 | | 2 | | | 3 | | 4 | | 5 | | 6 | | 7 | | | 8 | | | 9 | | 10 | |  |
|  |  |  |  |  |  |  | |  |  |  |  |  |  |  |  |  |  | |  |  | |  |  |  |  |  |
|  |  | |  | |  | | |  | |  | |  | |  | |  | | |  | | |  | |  | |  |

Overall, how bothersome to you is ____________ ?

| Not bothersome  at all | | | | |  | |  | | | | | | | | | | |  | | | Extremely  bothersome | | | | | |
| --- | --- | --- | --- | --- | --- | --- | --- | --- | --- | --- | --- | --- | --- | --- | --- | --- | --- | --- | --- | --- | --- | --- | --- | --- | --- | --- |
| ▼ | | | | |  | |  | | | | | | | | | | |  | | | ▼ | | | | | |
|  | 0 | | 1 | | 2 | | | 3 | | 4 | | 5 | | 6 | | 7 | | | 8 | | | 9 | | 10 | |  |
|  |  |  |  |  |  |  | |  |  |  |  |  |  |  |  |  |  | |  |  | |  |  |  |  |  |
|  |  | |  | |  | | |  | |  | |  | |  | |  | | |  | | |  | |  | |  |

Overall, how bothersome to you is ____________ ?

| Not bothersome  at all | | | | |  | |  | | | | | | | | | | |  | | | Extremely  bothersome | | | | | |
| --- | --- | --- | --- | --- | --- | --- | --- | --- | --- | --- | --- | --- | --- | --- | --- | --- | --- | --- | --- | --- | --- | --- | --- | --- | --- | --- |
| ▼ | | | | |  | |  | | | | | | | | | | |  | | | ▼ | | | | | |
|  | 0 | | 1 | | 2 | | | 3 | | 4 | | 5 | | 6 | | 7 | | | 8 | | | 9 | | 10 | |  |
|  |  |  |  |  |  |  | |  |  |  |  |  |  |  |  |  |  | |  |  | |  |  |  |  |  |
|  |  | |  | |  | | |  | |  | |  | |  | |  | | |  | | |  | |  | |  |

Overall, how bothersome to you is ____________ ?

| Not bothersome  at all | | | | |  | |  | | | | | | | | | | |  | | | Extremely  bothersome | | | | | |
| --- | --- | --- | --- | --- | --- | --- | --- | --- | --- | --- | --- | --- | --- | --- | --- | --- | --- | --- | --- | --- | --- | --- | --- | --- | --- | --- |
| ▼ | | | | |  | |  | | | | | | | | | | |  | | | ▼ | | | | | |
|  | 0 | | 1 | | 2 | | | 3 | | 4 | | 5 | | 6 | | 7 | | | 8 | | | 9 | | 10 | |  |
|  |  |  |  |  |  |  | |  |  |  |  |  |  |  |  |  |  | |  |  | |  |  |  |  |  |
|  |  | |  | |  | | |  | |  | |  | |  | |  | | |  | | |  | |  | |  |

Overall, how bothersome to you is ____________ ?

| Not bothersome  at all | | | | |  | |  | | | | | | | | | | |  | | | Extremely  bothersome | | | | | |
| --- | --- | --- | --- | --- | --- | --- | --- | --- | --- | --- | --- | --- | --- | --- | --- | --- | --- | --- | --- | --- | --- | --- | --- | --- | --- | --- |
| ▼ | | | | |  | |  | | | | | | | | | | |  | | | ▼ | | | | | |
|  | 0 | | 1 | | 2 | | | 3 | | 4 | | 5 | | 6 | | 7 | | | 8 | | | 9 | | 10 | |  |
|  |  |  |  |  |  |  | |  |  |  |  |  |  |  |  |  |  | |  |  | |  |  |  |  |  |
|  |  | |  | |  | | |  | |  | |  | |  | |  | | |  | | |  | |  | |  |

Overall, how bothersome to you is ____________ ?

| Not bothersome  at all | | | | |  | |  | | | | | | | | | | |  | | | Extremely  bothersome | | | | | |
| --- | --- | --- | --- | --- | --- | --- | --- | --- | --- | --- | --- | --- | --- | --- | --- | --- | --- | --- | --- | --- | --- | --- | --- | --- | --- | --- |
| ▼ | | | | |  | |  | | | | | | | | | | |  | | | ▼ | | | | | |
|  | 0 | | 1 | | 2 | | | 3 | | 4 | | 5 | | 6 | | 7 | | | 8 | | | 9 | | 10 | |  |
|  |  |  |  |  |  |  | |  |  |  |  |  |  |  |  |  |  | |  |  | |  |  |  |  |  |
|  |  | |  | |  | | |  | |  | |  | |  | |  | | |  | | |  | |  | |  |

Overall, how bothersome to you is ____________ ?

| Not bothersome  at all | | | | |  | |  | | | | | | | | | | |  | | | Extremely  bothersome | | | | | |
| --- | --- | --- | --- | --- | --- | --- | --- | --- | --- | --- | --- | --- | --- | --- | --- | --- | --- | --- | --- | --- | --- | --- | --- | --- | --- | --- |
| ▼ | | | | |  | |  | | | | | | | | | | |  | | | ▼ | | | | | |
|  | 0 | | 1 | | 2 | | | 3 | | 4 | | 5 | | 6 | | 7 | | | 8 | | | 9 | | 10 | |  |
|  |  |  |  |  |  |  | |  |  |  |  |  |  |  |  |  |  | |  |  | |  |  |  |  |  |
|  |  | |  | |  | | |  | |  | |  | |  | |  | | |  | | |  | |  | |  |

Overall, how bothersome to you is ____________ ?

| Not bothersome  at all | | | | |  | |  | | | | | | | | | | |  | | | Extremely  bothersome | | | | | |
| --- | --- | --- | --- | --- | --- | --- | --- | --- | --- | --- | --- | --- | --- | --- | --- | --- | --- | --- | --- | --- | --- | --- | --- | --- | --- | --- |
| ▼ | | | | |  | |  | | | | | | | | | | |  | | | ▼ | | | | | |
|  | 0 | | 1 | | 2 | | | 3 | | 4 | | 5 | | 6 | | 7 | | | 8 | | | 9 | | 10 | |  |
|  |  |  |  |  |  |  | |  |  |  |  |  |  |  |  |  |  | |  |  | |  |  |  |  |  |
|  |  | |  | |  | | |  | |  | |  | |  | |  | | |  | | |  | |  | |  |

Overall, how bothersome to you is ____________ ?

| Not bothersome  at all | | | | |  | |  | | | | | | | | | | |  | | | Extremely  bothersome | | | | | |
| --- | --- | --- | --- | --- | --- | --- | --- | --- | --- | --- | --- | --- | --- | --- | --- | --- | --- | --- | --- | --- | --- | --- | --- | --- | --- | --- |
| ▼ | | | | |  | |  | | | | | | | | | | |  | | | ▼ | | | | | |
|  | 0 | | 1 | | 2 | | | 3 | | 4 | | 5 | | 6 | | 7 | | | 8 | | | 9 | | 10 | |  |
|  |  |  |  |  |  |  | |  |  |  |  |  |  |  |  |  |  | |  |  | |  |  |  |  |  |
|  |  | |  | |  | | |  | |  | |  | |  | |  | | |  | | |  | |  | |  |

Treatment Decisions

[Duration: 10/60 minutes]

| ***Treatment*** |
| --- |

***Treatment for Neuropathic Pain***

1. Is there anything that makes your pain feel better?
   1. How do you know when it gets better? What changes?
2. When did you start treating your pain?
   1. How do you treat your neuropathic pain?

| ***Note to Interviewer****: Probe for the following treatment options* ***ONLY*** *if they have not been discussed at this point* |
| --- |

- - 1. Physical therapy
    2. Adjunctive therapies (spinal manipulation, hypnosis, acupuncture, etc.)
    3. Psychotherapy and counseling
    4. Opioids
    5. Antidepressants
    6. Muscle Relaxants
    7. Anticonvulsants
    8. Topical Analgesics (lidocaine patch, ketamine gel, capsaicin)

1. How has your treatment plan for your neuropathic pain changed over time?

***Treatment Decisions***

1. What are your goals of treatment? (Listen for: Maximizing function, limiting disability, improving symptoms?)
2. What factors were you considering when you started treatment for your pain?
   - ***Probe:*** *Your family, career, daily activities, effects on mood?*
3. How do you communicate with your doctor about treatment?
   - How often do you communicate with your doctor?
   - When do you communicate with your doctor about your pain?
